# Neurophysiologic effects of spinal manipulation in patients with chronic low back pain

**DOI:** 10.1186/1471-2474-12-170

**Published:** 2011-07-22

**Authors:** Brian C Clark, David A Goss, Stevan Walkowski, Richard L Hoffman, Andrew Ross, James S Thomas

**Affiliations:** 1 Ohio Musculoskeletal and Neurological Institute (OMNI), Ohio University, 236 Irvine Hall, Athens, OH 45701, USA; 2 Department of Biomedical Sciences, Ohio University Heritage College of Osteopathic Medicine, 228 Irvine Hall, Athens, OH 45701, USA; 3 Department of Family Medicine, Ohio University Heritage College of Osteopathic Medicine, Grosvenor Hall, Athens, OH 45701, USA; 4 School of Rehabilitation and Communication Sciences, Grover Center, Ohio University, Athens, OH 45701 USA

**Keywords:** Spinal manipulation, manual therapies, low back pain, muscle, stretch reflex, transcranial magnetic stimulation, chiropractic, osteopathic, audible release

## Abstract

**Background:**

While there is growing evidence for the efficacy of SM to treat LBP, little is known on the mechanisms and physiologic effects of these treatments. Accordingly, the purpose of this study was to determine whether SM alters the amplitude of the motor evoked potential (MEP) or the short-latency stretch reflex of the erector spinae muscles, and whether these physiologic responses depend on whether SM causes an audible joint sound.

**Methods:**

We used transcranial magnetic stimulation to elicit MEPs and electromechanical tapping to elicit short-latency stretch reflexes in 10 patients with chronic LBP and 10 asymptomatic controls. Neurophysiologic outcomes were measured before and after SM. Changes in MEP and stretch reflex amplitude were examined based on patient grouping (LBP vs. controls), and whether SM caused an audible joint sound.

**Results:**

SM did not alter the erector spinae MEP amplitude in patients with LBP (0.80 ± 0.33 vs. 0.80 ± 0.30 μV) or in asymptomatic controls (0.56 ± 0.09 vs. 0.57 ± 0.06 μV). Similarly, SM did not alter the erector spinae stretch reflex amplitude in patients with LBP (0.66 ± 0.12 vs. 0.66 ± 0.15 μV) or in asymptomatic controls (0.60 ± 0.09 vs. 0.55 ± 0.08 μV). Interestingly, study participants exhibiting an audible response exhibited a 20% decrease in the stretch reflex (p < 0.05).

**Conclusions:**

These findings suggest that a single SM treatment does not systematically alter corticospinal or stretch reflex excitability of the erector spinae muscles (when assessed ~ 10-minutes following SM); however, they do indicate that the stretch reflex is attenuated when SM causes an audible response. This finding provides insight into the mechanisms of SM, and suggests that SM that produces an audible response may mechanistically act to decrease the sensitivity of the muscle spindles and/or the various segmental sites of the Ia reflex pathway.

## Background

Low back pain (LBP) is one of the most common reasons for seeking medical care and accounts for over 3.7 million physician visits per year in the United States alone. Ninety percent of adults will experience LBP in their lifetime, 50% will experience recurrent back pain, and 10% will develop chronic pain and related disability [1-4]. According to the most recent national survey more than 18 million Americans over the age of 18 years received manipulative therapies in 2007 at a total annual out of pocket cost of $3.9 billion with back pain being the most common clinical complaint of these individuals [5].

Over the past decade there has been growing scientific evidence supporting the clinical efficacy [6-10] and effectiveness [11,12] of manual therapies in treating LBP. While clinical evidence supporting the efficacy and effectiveness of manual therapies has emerged, less scientific evidence has been offered to explain the effects and mechanisms underlying these treatments. The lack of a mechanistic underpinning hinders acceptance by the wider scientific and health-care communities, and it also limits the development of rational strategies for using manipulative therapies.

Many scientists and clinicians have long-postulated that manual therapies exert their biologic effects on segmental components of the central nervous system (e.g., supraspinal, spinal, etc.) [13-22]. For example, more than 25 years ago it was noticed that deep somatic or visceral pain leads to increases in muscle tone/spasm in the surrounding musculature [23], and many authors have speculated that an increased stretch reflex gain underlies the increased muscle tone in painful muscles as is commonly observed in LBP [16-18,24]. Interestingly, the limited animal [25] or human [26] data that exists does not support this common clinical assertion. However, the reflex activity of human back muscles has received little attention [26-30], and to our knowledge, no studies have quantified the effects of spinal manipulation (SM; the most common manual therapy used to treat LBP [31,32]) on the stretch reflex excitability of the low back muscles despite this being such a commonly touted mechanism of action.

The scientific understanding of the neurophysiologic characteristics of the human low back muscles has historically been hindered by the lack of experimental techniques to examine these muscles' function *in vivo*. However, in recent years innovative advancements in neurophysiologic assessment techniques--such as transcranial magnetic stimulation (TMS) to elicit motor evoked potentials (MEP) [15,33,34] and mechanically elicited stretch reflexes [26,29,33]-- have begun to be applied to the study of the human lumbar musculature. In this study we utilized these neurophysiologic techniques to determine the effects of a single high-velocity low-amplitude SM thrust on corticospinal and stretch reflex excitability in patients with chronic LBP and in asymptomatic controls. Specifically, we quantified the effects of SM on the motor evoked potential (MEP) and short-latency stretch reflex amplitude of the erector spinae muscles. In addition to determining whether the MEP and stretch reflex amplitude were altered in individuals with and without LBP, we also examined whether these physiologic responses depended on whether the spinal manipulation caused an audible sound from the joint (i.e., the pop or cracking sound that one often associates with joint manipulations). The role of the audible response in determining treatment effects has long been a matter of intense debate. Some studies have previously reported that an audible response is not necessary to improve clinical outcomes [35,36], however, some have reported increased joint laxity, motion and gapping following manipulation that results in an audible sound [37,38] but few studies have investigated if the physiologic response is dependent on the manipulation causing an audible joint sound.

## Methods

### General Overview of the Experimental Design

In this case-control study we wished to answer the following questions: 1) does a single SM alter corticospinal excitability of the erector spinae muscles in patients with chronic LBP or in asymptomatic controls; 2) does a single SM alter the excitability of the Ia reflex pathway of the erector spinae muscles in patients with chronic LBP or in asymptomatic controls; and 3) Do the changes in corticospinal or Ia reflex pathway excitability vary depending on whether an audible response occurs during SM? To address these questions we recruited ten patients with chronic LBP and ten healthy individuals without LBP. A baseline neurophysiologic testing session was conducted using TMS to quantify MEP amplitude (as an index of corticospinal excitability), and electromechanical tapping of the lumbar paraspinal muscles to quantify short-latency stretch reflex amplitude (as an index of Ia reflex pathway excitability). Subsequently, a single high-velocity low-amplitude SM thrust was delivered to the lumbar spine, and ~ 10-minutes later the aforementioned neurophysiologic testing session was repeated. During the SM procedure the treating physician and at least one other researcher took special care to listen during the SM procedure, confer with each other, and document the study participants who exhibited an audible response. This documentation occurred immediately following the SM procedure, but the subject was not consulted regarding whether they heard an audible response nor were they aware that we were documenting these responses. Data were examined to determine if the MEP or stretch reflex amplitude changed following SM between patient groups (LBP and controls), and those who exhibited an audible response (audible response vs. no audible response).

### Study Participants

Ten patients (5 men, 5 women) with chronic LBP (defined as > 12 weeks) were recruited from advertisements in the local community to participate in this study, and ten healthy individuals without back pain were matched on a case-by-case basis for sex and for within 10% on age and body mass index. To qualify for study participation, chronic LBP patients had to have experienced LBP for at least 12-weeks, and to have previously sought medical care, chiropractic care or physical therapy for treatment of their LBP. Individuals were excluded if they had a history of spinal surgery, other orthopedic or neurological impairments, spinal fractures, tumors, arthroplasties, osteoporosis, cardiopulmonary disorders, or severe osteoarthritis. Study participants were also excluded if they were currently using narcotics or muscle relaxants for pain, were pregnant, exhibited frank neurologic signs, or had a body mass index greater than 32 kg/m^2^, had clinical depression, if they reported unexplained weight loss or an elevated temperature, had received any manual therapy intervention in the past 1-month, or if they had pending litigation related to an episode of LBP or were receiving disability. Lastly, study participants were excluded if they were taking medications known to influence TMS parameters [39] or had any conditions that are contraindicated for exposure to a magnetic field [40]. The asymptomatic controls were matched for age, sex, and body mass index to the LBP patients. The control subjects were recruited by word of mouth and electronic mailing to the broader university community. To be included in the study, the asymptomatic control subjects had to report no history of LBP and rate their current LBP a zero on a 0-10 visual analog scale. The Institutional Review Board at Ohio University approved the study protocol, and study participants gave their written informed consent prior to participation.

### Characterization of Low Back Pain

To characterize the LBP of the patient population, subjects were asked to 1) rate their *usual *LBP on a 0-10 visual analog scale, 2) rate their *current *LBP on a 0-10 visual analog scale, 3) rate their *lifestyle change *imposed by their LBP on a 0-10 visual analog scale, and complete the Roland Morris Disability Questionnaire [41], and the Tampa Scale for Kinesiophobia [42]. These surveys were completed at the start of the testing session (prior to undergoing any of the physiological testing or the SM procedure).

### History and Physical Examination

Interested participants completed a standard medical history form during the inclusion and exclusion screening process. On the day of testing, a physical examination was also completed. Here, subjects were assessed in the standing, seated, and supine positions to evaluate for the presence of somatic dysfunction in the thoracic, lumbar, sacral, or pelvic regions. This involved a palpatory screening assessment for alterations in tissue texture change and alterations in normal regional motion, followed by more detailed palpatory diagnostic procedures designed to localize the specific dysfunctional spinal segment or segments in each of the subjects. These palpatory procedures utilized normal landmark identification in the named regions and motion testing at a vertebral segmental level to determine the extent and severity of motion restriction along with increases in tissue hypertonicity and/or tenderness to palpation.

### Electrical Recordings

Electrical signals were recorded bilaterally from the erector spinae (ES) muscles as we have previously described [33]. In brief, bipolar differential surface electrodes (Ag-AgCl, potential sensitive area of 22-mm; 2015 Nikomed Trace1, Hudson Valley, PA) were placed parallel to the spine's long axis with one electrode positioned at L2 and the other positioned 6-cm directly below (6-cm center-to-center interelectrode distance). These electrodes were placed ~ 2-3 cm lateral of the spine over the belly of the erector spinae muscles. A reference electrode was placed over the anterior superior iliac spine. The electromyogram (EMG) signals were amplified (1000×), band pass filtered (10-500 Hz), and sampled at 5,000 Hz using a 16-bit data acquisition system (MP150, BioPac Systems Inc.). Electrodes were left in place throughout the duration of the testing session.

### Transcranial Magnetic Stimulation

**Figure 1:**
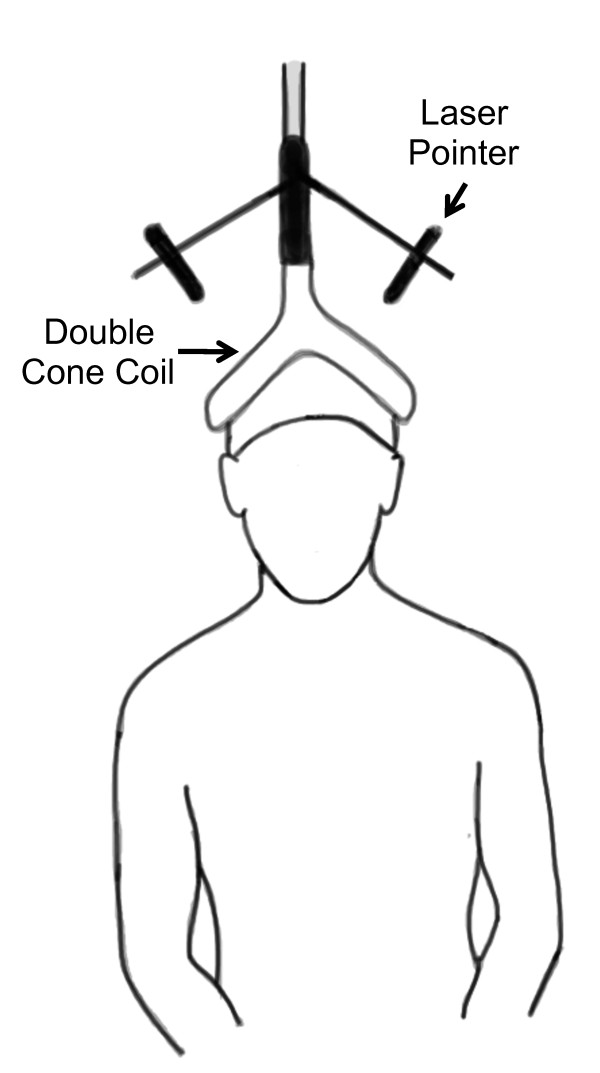
**The experimental setup for performing transcranial magnetic stimulation (TMS; left) to evoke motor evoked potentials (MEP; right) from the lumbar erector spinae muscles**. SA=Stimulus artifact.

TMS pulses were delivered at the vertex of the skull similar to our previous description to elicit motor evoked potentials in the lumbar erector spinae muscles (Figure 1) [33]. Prior to performing TMS, anthropometric measurements of the skull were taken to identify the vertex while the subject wore a lycra cap. Here, we used an anthropometer (Model 01290 Large Anthropometer, Lafayette Instruments, Lafayette, IN) to identify the vertex as defined by the intersection of the skull in the sagittal (between the nasion and inion) and coronal (between the tragus) planes. The center of a custom-modified 110-mm double cone coil with a laser attachment system (The Magstim Co. Ltd., Whitland, England) was positioned over the vertex to stimulate the underlying cortical structures. Single-pulse stimuli were delivered using a Magstim 200^2 ^(The Magstim Co. Ltd., Whitland, England) magnetic stimulator with the direction of current flowing from an anterior-to-posterior direction. During TMS, study participants were asked to sit with an upright posture while their hands rested in their lap. They were seated in a swivel-base chair with the thigh at 90-degrees relative to the trunk, the lower leg at ~ 45 degrees relative to the thigh, and the lumbar spine in a neutral posture. Care was taken across all trials to ensure that the same posture was maintained.

We began the TMS protocol by delivering a series of three single pulses at increasing intensities (40, 60 and 80% of the maximum stimulator output) to allow the subject to gradually become acquainted with TMS. Next, the pulse intensity was increased to 100% of maximum stimulator output and a series of ten pulses separated by 15 seconds were delivered, and the peak-to-peak amplitude of the MEPs calculated and averaged. During all analyses we visually analyzed the EMG traces to ensure that the TMS responses did not occur in temporal relation with the electrocardiogram signals (to avoid interference with the EMG signals), and in the rare instance that this did occur the MEP was excluded from analysis (this occurred in no more than one trial per a given subject). Following the baseline TMS testing protocol the stretch reflex testing protocol was performed and study participants then received SM. Ten minutes after the study participant received SM the TMS and stretch reflex protocols were then repeated.

### Stretch Reflex

**Figure 2:**
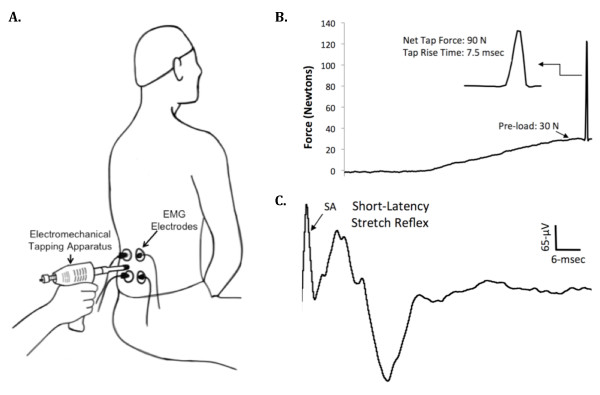
****A**. The experimental setup for evoking short-latency stretch reflexes from the lumbar paraspinal muscles**. **B**. The tip of the electromechanical tapping apparatus was gradually pressed into the tissue to a pre-load of 30-Newtons was reached at which time the device delivered a rapid mechanical tap to the muscle with a net force of 90-Newtons. **C**. Representative examples of a short-latency stretch reflex recorded from the lumbar paraspinal muscles in response to a mechanical tap. SA= Stimulus artifact.

When a muscle is rapidly stretched, a short-latency stretch reflex is elicited due to the excitation of the receptive endings of the Ia afferent fibers within the muscle spindles [43]. To quantify the erector spinae muscle stretch reflex responsiveness we determined the EMG activity of the muscle in response to mechanical tapping as we have previously described (Figure 2) [33]. During the assessment of the stretch reflex study participants were seated in the same chair and maintained the same posture as we described above for the TMS testing protocol. To elicit lumbar paraspinal muscle stretch reflexes we used a custom-modified version of an electromechanical tapping device (ArthoStim^®^, IMPAC Inc. Salem, OR) with a 1-cm diameter hard rubber tip. Using this device a single mechanical tap was delivered to the belly of the left and right erector spinae muscles at the L3 vertebral level between the EMG electrodes (Figure 2). To elicit the stretch reflexes the electromechanical prodder was placed against the skin and the pressure applied to the low back tissue was gradually increased to 30-Newtons at which time the device delivered a mechanical tap to the muscle with a net force of 90-Newtons (Figure 2). These mechanical taps evoked a short-latency stretch reflex that occurred ~ 5-7 msec following the cessation of the mechanical tap, which is consistent with the expected latency value for a monosynaptic stretch reflex for the lumbar paraspinal muscles (Figure 2C) [29]. A total of ten short-latency stretch reflexes were elicited on each side by tapping the muscle with at least 10 seconds separating each reflex response. The corresponding EMG responses were recorded, and the peak-to-peak amplitude of the reflex responses was averaged to assess stretch reflex excitability. A mark was made with indelible ink on the skin to ensure tapping was applied to the same site. During all analyses, special care was taken to ensure that the stretch reflex responses did not occur in temporal relation with the electrocardiogram signals (to avoid interference with the EMG signals), and in the rare instance that this did occur the trial was excluded from analysis (this occurred in no more than one trial per a given subject).

### Spinal Manipulation

**Figure 3:**
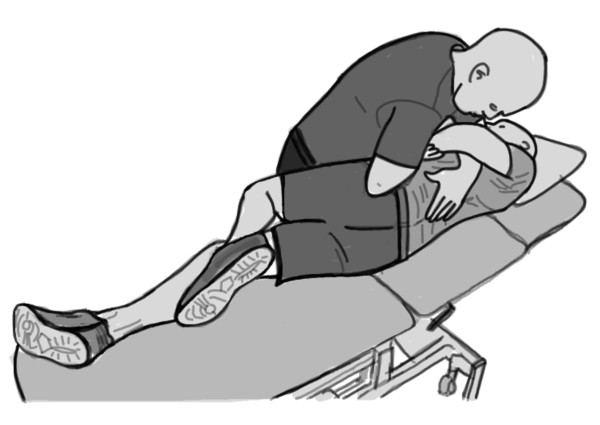
**Schematic illustration of the high-velocity low-amplitude spinal manipulation technique**.

We used a long-lever rotary spinal manipulation technique with the subject in a side-lying position (Figure 3). Study participants were positioned in a lateral recumbent or side-lying position with the superior or free hip and knee flexed and adducted across the midline. During the procedure the clinician stabilized the subject's free leg with their own leg while holding the participant's superior shoulder and the manipulative force was applied with the clinician's forearm resting on the pelvis. The rotatory thrust on the pelvis directed at a localized lumbar segment was delivered by a quick, short, controlled movement of the shoulder and arm combined with a slight body drop. The vertebral segments chosen for manipulation were chosen based on palpatory structural diagnosis assessing for position and motion restriction consistent with standard osteopathic structural diagnostic procedures. The manipulation force applied was localized to the dysfunctional vertebral segment utilizing alignments of force vectors secondary to subject positioning.

### Statistical Analysis

Mixed-model analysis of variance techniques were utilized to determine the effect of the independent variables (i.e., within-subjects factor: time (pre- and post-SM; between-subjects factors: patient group (LBP and asymptomatic controls); audible response group (audible response and non-audible response)) on the dependent variables (MEP amplitude and short-latency stretch reflex amplitude). Initially, the left and right side erector spinae data were subjected to these analyses; however, because no side-to-side differences were observed the data from each side were subsequently averaged and are presented averaged herein for the sake of clarity. For all analyses, a preset alpha-level of significance equal to 0.05 was required for statistical significance, and significant main effects or interaction terms were followed up with Sidak post hoc tests. The SPSS statistical package (version 18.0, Chicago, IL) was used for data analysis. Data are presented as means ± SE, unless otherwise stated. Sample size for the present study was based on our previously observed effect sizes associated with reductions in muscle activity levels following other manipulative therapies [13]. Eta-squared (η^2^) estimates of effect size are also reported to provide the reader insight on the magnitude of effect of SM.

## Results

### Study Participants' Descriptive Statistics

There were no differences in the mean age of the LBP patients in comparison to the control subjects (23.7 ± 6.1 and 22.9 ± 1.9 years; p = 0.73). There were no differences in the mean height of the LBP patients in comparison to the control subjects (was 171.7 ± 13.2 and 174.5 ± 8.4 cm; p = 0.58). There were no differences in the mean weight of the LBP patients in comparison to the control subjects (67.9 ± 11.5 and 70.3 ± 11.8 kg; p = 0.64). Lastly, there were no differences in the mean body mass index of the LBP patients in comparison to the control subjects (23.0 ± 2.3 and 23.0 ± 2.8 kg/m^2^; p = 0.96). Using a 0-10 visual analog scale, the chronic LBP patients rated their usual LBP as 4.0 ± 1.2, their current LBP as 2.6 ± 1.6, and their lifestyle change imposed from their LBP as 3.9 ± 3.1. Additionally, they reported having LBP for a mean duration of 3.2 ± 3.1 years, scored 5.9 ± 4.3 on the Roland Morris Disability Questionnaire, [41] and scored 33.5 ± 6.5 on the Tampa Scale for Kinesiophobia [42].

### Effects of spinal manipulation on erector spinae motor evoked potential amplitude in patients with chronic LBP and in asymptomatic controls

**Figure 4:**
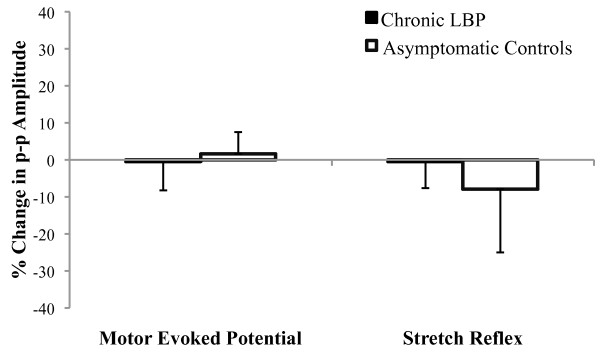
**Spinal manipulation did not alter the amplitude of the motor evoked potential (left) or short-latency stretch reflex (right) recorded from the erector spinae muscles in patients with chronic low back pain (LBP) or in asymptomatic controls**.

SM did not alter the erector spinae MEP amplitude in patients with LBP (0.80 ± 0.32 to 0.80 ± 0.30 μV) or in asymptomatic controls (0.56 ± 0.09 to 0.57 ± 0.06 μV) (Figure 4; group main effect: p = 0.48, η^2 ^= 0.03; time main effect: p = 0.61, η^2 ^= 0.02; time × group interaction: p = 0.62, η^2 ^= 0.01).

### Effects of spinal manipulation on erector spinae short-latency stretch reflex amplitude in patients with chronic LBP and in asymptomatic controls

SM did not alter the erector spinae stretch reflex amplitude in patients with LBP (0.66 ± 0.12 to 0.66 ± 0.15 μV) or in asymptomatic controls (0.60 ± 0.09 to 0.55 ± 0.08 μV) (Figure 4; group main effect: p = 0.41, η^2 ^= 0.04; time main effect: p = 0.92, η^2 ^< 0.01; time × group interaction: p = 0.90, η^2 ^< 0.01).

### Effects of spinal manipulation on erector spinae motor evoked potential and short-latency stretch reflex amplitude in which the spinal manipulation did and did not produce an audible joint sound

**Figure 5:**
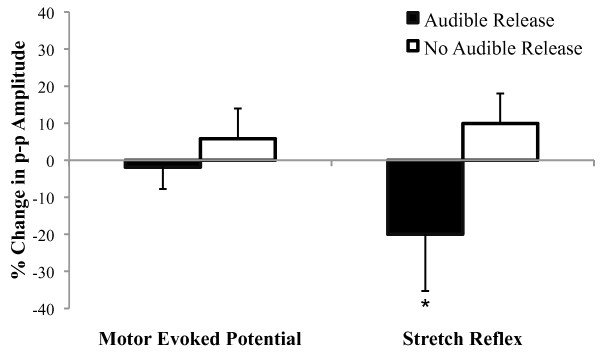
**Spinal manipulation did not alter the amplitude of the motor evoked potential (left) recorded from the erector spinae muscles in individuals who exhibited an audible response in response to spinal manipulation or in those who did not.** Interestingly, spinal manipulation did reduce the amplitude of the short-latency stretch reflex in individuals who exhibited an audible response in response to spinal manipulation when compared to those who did not (*time × group interaction: p = 0.05; η^2 ^= 0.20).

Eleven study participants exhibited an audible joint sound to SM (5 participants with LBP and 6 controls), whereas nine did not (5 participants with LBP, and 4 controls). SM did not alter the erector spinae MEP amplitude in individuals who exhibited an audible response (0.83 ± 0.09 to 0.81 ± 0.08 μV) or in those who did not (0.50 ± 0.02 to 0.53 ± 0.01 μV) (Figure 5; time main effect: p = 0.88, η^2 ^< 0.01; time × group interaction: p = 0.58, η^2 ^= 0.02). There were no notable differences in the magnitude of change in the MEPs between the LBP patients (2% increase) and the control subjects (7% decrease) among those exhibiting an audible response during SM. Interestingly, erector spinae stretch reflex was reduced 19.2% when SM caused an audible joint sound (0.54 ± 0.02 to 0.43 ± 0.01 μV), whereas when SM did not cause an audible joint sound there was a 9.7% increase (0.73 ± 0.05 to 0.81 ± 0.05 μV) (Figure 5; time × group interaction: p = 0.05; η^2 ^= 0.20). There were no notable differences in the magnitude of change in the stretch reflex between the LBP patients (16% decrease) and the control subjects (22% decrease) among those exhibiting an audible response during SM.

## Discussion

It has long-been suggested that SM exerts beneficial effects by affecting the nervous system [13-22]; however, to date few studies have examined these claims in humans with LBP. As such, utilizing advanced neurophysiologic assessment techniques to investigate the effects of a commonly used-- but poorly understood-- treatment for LBP is particularly innovative. The most novel findings of the present study are: i) that a single spinal manipulation does not systematically alter corticospinal or the short-latency stretch reflex excitability of the erector spinae muscles in patients with chronic LBP or asymptomatic controls (at least when assessed ~ 10-min following manipulation), and ii) that only when spinal manipulation induces an audible joint sound the erector spine short-latency stretch reflex is attenuated. Below we will discuss these findings in the context of understanding the physiological effects of spinal manipulation.

**Figure 6:**
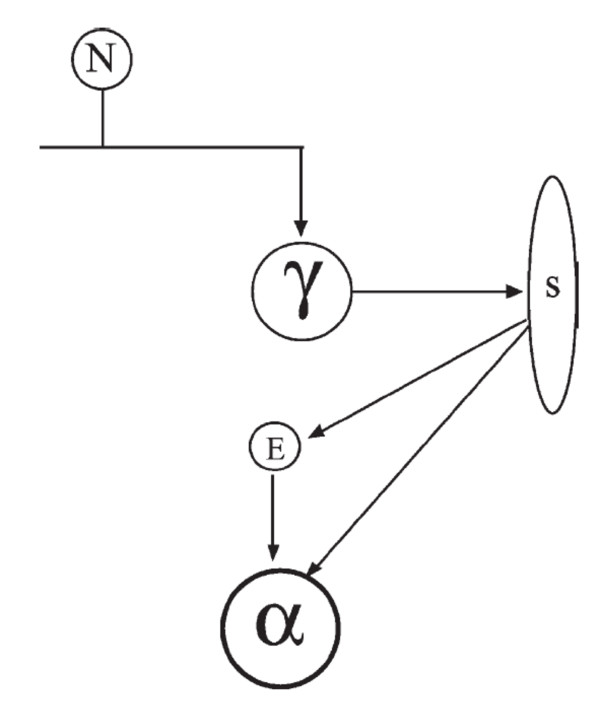
**A commonly proposed neural pathway suggested to form the basis of a pain-spasm-pain cycle**. Specifically, it has been suggested that feedback of nociceptive afferents (N) on the gamma-motorneurons (γ) will increase the sensitivity of the muscle spindles (S) to stretch, which results in direct and indirect excitatory input (E) to the alpha-motorneurons (α) that will subsequently increase muscle activation. *Reprinted from van Dieen et al., J Electromyogr Kineesiol. 13(4): 333-351, 2003.*

A recent review of chronic LBP provides evidence for two prominent pain theories [44]. One of these pain theories, the pain-spasm-pain model of chronic LBP, suggests that pain leads to muscular hyperactivity (spasm), which in turn causes pain. One of the neural pathways of the pain-spasm-pain cycle posits that a hyperactive spinal stretch reflex forms the basis of the cycle (Figure 6). Specifically, it has been suggested that feedback of nociceptive afferents on the gamma-motorneurons will increase the sensitivity of the muscle spindles to stretch, which results in excitatory input to the alpha-motorneurons that will subsequently increase muscle activation (for review see [44]). While several studies suggest there is no increase in spindle sensitivity with low back pain or noxious stimulation of paraspinal tissues [25,26], many authors have still postulated that SM functions *via *the pain-spasm-pain model by reducing the underlying nociceptive stimulus and consequently attenuating the stretch reflex, with the end organ effect being an overall reduction in muscle activity [45-50]. Indeed, several studies have noted reduced paraspinal voluntary EMG amplitude following SM of individuals with LBP [51-54], and we recently reported that a combination of manual therapies (incorporating both SM and soft-tissue techniques) normalizes side-to-side differences in the activation patterns of trunk muscles of individuals with sub-acute LBP, as determined by muscle functional magnetic resonance imaging [13].

In the present study we did not quantify changes in muscle activity following SM, but rather assessed the effects of SM on the evoked short-latency stretch reflex amplitude. Although our observation of no pre- versus post-manipulation difference in patients with chronic LBP or asymptomatic controls suggested that SM did not systematically alter the short-latency stretch reflex, we did observe a significant decrease in the short-latency stretch reflex when data were analyzed based on whether SM resulted in an audible joint sound. Many clinicians routinely consider the success of a thrust manipulation technique based on the presence or absence of an audible response. While some evidence suggests that an audible response is not associated with improved clinical outcomes [35,36], there are differences in joint laxity and motion when an audible pop is associated with the manipulation [37]. This may reflect the successful and rapid separation of the joint surfaces resulting in cavitation and an audible response. It has been hypothesized that the rapid stretch of the periarticular muscles and connective tissue associated with SM causes the reduction in spinal reflexes [17,18]; however, to our knowledge no previous studies have reported differential physiologic effects dependent upon whether SM results in an audible response. Thus, our finding that SM alters the short-latency stretch reflex--a critical component of the pain-spasm-pain model of LBP (Figure 6)--only when an audible response occurs is novel. As stated before, the short-latency stretch reflex occurs in response to a muscle being rapidly stretched, which excites the Ia afferent fibers within the muscle spindles [55]. This observation suggests that when SM results in an audible response it mechanistically acts by down-regulating the sensitivity of the muscle spindles and/or the various other segmental sites of the Ia stretch reflex pathway. It is also possible that the change in reflex activity associated with subjects having an audible release during SM may relate to gapping in the joint surfaces, as it was recently shown that vertebral segments that cavitated during SM gapped (separated) more than those that did not [38]. This greater joint gapping could result in the break-up of small adhesions present even in normal joints, or due to increased muscle or connective tissue tension surrounding those joints, before SM. Consequently, SM that results in an audible response may conceivably function to restore greater motion to a vertebral segment (as opposed to SM that does not result in an audible response), and this biomechanical effect could result in subsequent changes in reflex activity as we observed.

We did not observe changes in MEP amplitude following SM in patients with LBP, asymptomatic controls, or when data were grouped according to whether an audible response was observed. When a single pulse transcranial magnetic stimulation stimuli is applied to the motor cortex at an intensity above motor threshold, high-frequency indirect waves (I waves) are elicited in the corticospinal tract [56], which are modifiable by many mechanisms (i.e., glutatmate, GABA, acetylcholine, etc.) [39] that influence the amplitude of the MEP. Thus, our finding of no change in the MEP indicates that a single SM treatment in patients with chronic LBP does not alter global excitability of the corticospinal tract, at least when assessed ~ 10-min following the manipulative intervention. To date only one other study has examined the effects of SM on corticospinal excitability of the low back muscles using transcranial magnetic stimulation [15]. In this study Dishman and colleagues examined the effects of a single SM treatment on MEP amplitude in *asymptomatic *young adults, and observed a transient increase in the MEP following SM. The MEP facilitation was short-lived however-- as MEP amplitude was increased 10-secs following SM but had returned to baseline levels less than 20-seconds after SM. Thus, in the present we would have missed any short-term, transient effects that occurred as a result of SM. Additionally, in our work as well as that conducted by Dishman et al. it is possible that segmental changes in the nervous systems excitability (e.g., cortical level changes) may have been confounded by no change in or opposite changes in excitability at a different segmental level (e.g., spinal level changes) as the MEP amplitude elicited using *single-pulse *transcranial magnetic stimulation can be influenced at both the cortical and spinal levels. To more fully explore the effects of SM on cortico-cortical excitability it is suggested that future investigations utilize paired-pulse transcranial magnetic stimulation to measure intracortical facilitation and inhibition.

There are several limitations of the present study that should be mentioned. First, it should be noted that the present work was conducted in patients with mild-to-moderate chronic LBP and asymptomatic controls, and that these individuals only received a single high-velocity low-amplitude SM thrust with outcome measures assessed shortly after the manipulative treatment. As such, it is possible that i) SM may result in different physiologic responses in other populations (e.g., sub-acute LBP), ii) that a course of SM treatment may have a more pronounced effect (e.g., three weeks of SM twice per week), and/or iii) that greater or lesser effects may have been observed at various time points following manipulation. Additionally, we chose to study chronic LBP patients (as opposed to acute or sub-acute LBP patients) due to the staggering economic costs that are associated with chronic LBP and the fact that many patients with chronic LBP seek manipulation therapy as a treatment option for their LBP [5]. However, it is possible that the neurophysiologic responses may be different if other groups of LBP patients had been studied as patients with LBP symptom duration for < 16 days are reported to be more likely to respond favorably to SM [57]. Further, we cannot rule out the potential for a placebo effect to influence our observed reduction in the stretch reflex in individuals who exhibited an audible response to SM. However, with this stated, it seems unlikely that this finding was driven by a placebo effect as one would likely expect to observe a concomitant change in corticospinal excitability-- as a placebo effect would likely be assumed to have systemic effects (as opposed to having a local, selective effect on the stretch reflex only).

## Conclusion

In summary, this study examined the effects of spinal manipulation on the motor evoked potential and short-latency stretch reflex amplitudes of the erector spinae muscles in patients with chronic low back pain and asymptomatic controls. We did not observe changes in these outcomes in either group when assessed ~ 10-minutes following a single spinal manipulative thrust. Interestingly, when data were analyzed according to whether spinal manipulation caused an audible joint sound, regardless of patient group, we observed that study participants exhibiting an audible response exhibited a significant reduction in the short-latency stretch reflex. These findings suggest that a single SM treatment does not systematically alter corticospinal or stretch reflex excitability of the erector spinae muscles; however, they do indicate that the stretch reflex is attenuated when spinal manipulation causes an audible joint sound. This finding provides insight into the mechanism(s) of action of spinal manipulation, and suggests that spinal manipulation may mechanistically act by down regulating the gain of the muscle spindles and/or the various segmental sites of the Ia reflex pathway. Developing a better understanding of the physiologic effects of various manual therapies to treat low back pain will in the long-term assist in optimizing and developing strategic treatment strategies for specific patient populations with LBP.

## Competing interests

The authors declare that they have no competing interests.

## Authors' contributions

BC participated in the conception and design of the study, supervised the data collection, helped secure grant funding, and drafted the manuscript. DG, RH, and AR recruited and screened subjects and were actively involved in all data collection procedures. DG and SW performed the spinal manipulative procedures. JT participated in the conception and design of the study, supervised the data collection, and helped secure grant funding. All authors read and approved the final manuscript.

## Pre-publication history

The pre-publication history for this paper can be accessed here:

http://www.biomedcentral.com/1471-2474/12/170/prepub
